# Dengue shock syndrome complicated with acute liver failure and kidney injury, infective endocarditis, and deep vein thrombosis: a case report

**DOI:** 10.1186/s13256-018-1862-1

**Published:** 2018-10-30

**Authors:** Keshinie Samarasekara, Janake Munasinghe

**Affiliations:** National Hospital of Sri Lanka, E W Perera Mawatha, Colombo, 00700 Sri Lanka

**Keywords:** Dengue shock syndrome, Infective endocarditis, Deep vein thrombosis

## Abstract

**Background:**

Dengue fever is a mosquito-borne viral disease with a very high incidence in Southeast Asia. Most patients with dengue fever recover following a self-limiting febrile illness, while a small proportion may progress to develop severe disease with complications such as acute liver failure, acute kidney injury, and multiorgan failure. Secondary bacterial infections and thrombotic events are very rare.

**Case presentation:**

A 38-year-old previously healthy Sri Lankan woman from Colombo, Sri Lanka, presented with dengue shock syndrome leading to acute liver failure and kidney injury. She was managed with intravenously administered fluid resuscitation with close monitoring of her hemodynamic status, and hemodialysis. Her renal and liver functions and platelet count improved gradually, but the fever persisted and there was a neutrophil leukocytosis. A clinical examination and investigations to identify a focus of secondary infection revealed staphylococcal infective endocarditis. She was started on intravenously administered vancomycin, but as the response was poor the antibiotic was changed to intravenously administered linezolid, to which the response was good. She also developed right proximal femoral deep vein thrombosis, and was commenced on subcutaneous enoxaparin and warfarin. Enoxaparin was stopped after her international normalized ratio reached the desirable range, and warfarin was continued for 3 months.

**Conclusions:**

Dengue virus is known to cause endothelial dysfunction that allows bacteria to invade tissues, defective functioning and reduction in the number of cells of the immune system, and alteration of cytokines leading to immune dysregulation, predisposing patients to develop secondary bacterial infections. Evidently, patients with dengue fever who have prolonged fever (more than 5 days) and acute kidney injury are at high risk for concurrent bacteremia. Dengue virus interferes with the components of the anti-clotting pathway, such as thrombomodulin-thrombin-protein C complex. It also activates endothelial cells and increases the expression of procoagulant factors. These factors may predispose patients with dengue viral infections to develop thrombotic complications. Therefore it is important to be aware of the possibility of serious secondary bacterial infections occurring following dengue viral infections, especially in patients with prolonged fever and acute kidney injury, and to keep in mind that thrombotic events may occur as complications of dengue viral infections.

## Background

Dengue fever (DF) is a mosquito-borne viral disease caused by the dengue virus, which belongs to the family *Flaviviridae* and genus *Flavivirus* [1]. Sri Lanka is an island nation in Southeast Asia, with a population of around 21 million [2].

DF is endemic in Sri Lanka, and accounts for a large proportion of hospital admissions with acute fever. In the first half of 2017 (from 1 January to 7 July 2017), the Epidemiology Unit of the Ministry of Health, Sri Lanka reported 80,732 cases of DF, including 215 deaths. This is 4.3 fold higher than the average number of cases for the same period in the preceding 7 years. Approximately 43% of the cases of DF were reported from the Western Province and the most affected area with the highest number of reported cases was Colombo District [3].

Most patients recover following a self-limiting febrile illness, while a small proportion may progress to develop severe disease, characterized by plasma leakage and shock, with or without hemorrhage. Acute liver failure, acute kidney injury, and multiorgan failure are well-known complications of severe disease [1].

There are reported cases of staphylococcal superinfection or co-infection occurring in patients with dengue viral infections [4, 5]. However, there is only one reported case of infective endocarditis occurring in a patient with dengue viral infection [6].

Hemorrhagic manifestations are common in dengue, and thrombotic events are uncommon. However, there are case reports and a case series in the literature on the occurrence of deep vein thrombosis associated with dengue viral infection [7–9].

We report the case of a patient with dengue shock syndrome leading to acute liver failure and kidney injury, complicated with staphylococcal infective endocarditis and right proximal femoral deep vein thrombosis.

## Case presentation

A 38-year-old previously healthy Sri Lankan woman from Colombo, Sri Lanka presented to a teaching hospital on day 5 of an acute febrile illness. On admission to the medical ward, she was afebrile, with a pulse rate of 120 beats per minute and a blood pressure of 80/60 mmHg. She also had features of a right-sided pleural effusion on examination of her lungs, and an abdomen examination revealed tender hepatomegaly with free fluid.

The results of the investigations done on presentation were as follows: white blood cell count 3400/mm^3^ (neutrophils 45%, lymphocytes 43%); platelets 18,000/mm^3^; hemoglobin 11.7 g/dl; hematocrit 49.4%; blood picture – leukopenia, lymphocytosis, and thrombocytopenia suggestive of an acute viral infection; erythrocyte sedimentation rate 06 mm/hour; alanine aminotransferase 1360 U/l; aspartate aminotransferase 2450 U/l; alkaline phosphatase 185 U/l; total bilirubin 1.4 mg/dl; direct bilirubin 0.5 mg/dl; serum protein 5.7 g/dl; serum albumin 2.9 g/dl; prothrombin time 19 seconds; international normalized ratio 1.58; serum creatinine 4.6 mg/dl; serum sodium 143 mmol/l; and serum potassium 5.5 mmol/l.

A clinical diagnosis of possible dengue hemorrhagic fever with shock leading to acute liver and kidney injury was made based on the history, examination, investigations, and the very high incidence of DF in Colombo during the time of her presentation. It was confirmed subsequently with seroconversion of dengue immunoglobulin M (IgM) antibody test (enzyme linked immunosorbent assay) on day 7 of the illness.

She was managed with intravenously administered fluid resuscitation and close monitoring of her hemodynamic status. Following initial stabilization, hemodialysis was done via right-sided femoral venous access. By day 8 of the illness, her serum creatinine declined to 2.1 mg/dl, and the femoral venous catheter was removed as she no longer required hemodialysis.

Her liver functions and platelet count improved gradually. However, by day 10 of the illness, the fever was persistent and her white cell count rose to 13,500/mm^3^ with 73% neutrophils. A clinical examination to identify a focus of secondary infection revealed a systolic murmur best heard at the mitral area. There were no peripheral stigmata of infective endocarditis. There were no signs of infection at the site of previous femoral venous access.

A two-dimensional echocardiogram was performed and a 0.5 × 0.3 cm sized oscillating intracardiac mass was found attached to anterior mitral valve leaflet. One blood culture was positive for methicillin-resistant *Staphylococcus aureus* (MRSA), which was sensitive to vancomycin with minimum inhibitory concentration (MIC) 0.5 μg/ml and linezolid, but all the other blood cultures were negative. A diagnosis of possible infective endocarditis was made according to modified Duke criteria (one major criterion – presence of vegetation, and two minor criteria – single positive blood culture and fever).

She was started on intravenously administered vancomycin 1 g daily, to which the response was poor despite adequate trough levels. Therefore, the antibiotic was changed to intravenously administered linezolid 600 mg 12 hourly, to which the response was good. Linezolid was chosen according to the sensitivity pattern of the MRSA strain and due to unavailability of daptomycin or fifth-generation cephalosporins. A repeat two-dimensional echocardiogram was done after completing 2 weeks of antibiotics; there was no vegetation, which was confirmed with a transesophageal echocardiogram. Blood cultures were negative. However, the antibiotic was continued for a total duration of 4 weeks. Blood counts were monitored closely to detect cytopenia caused by linezolid.

On day 14 of the illness, she complained of right lower limb pain, and swelling of right lower limb was noted. A venous duplex of the lower limbs revealed right proximal femoral deep vein thrombosis.

She was commenced on subcutaneous enoxaparin 60 mg twice daily, and warfarin 5 mg daily. The dose of warfarin was adjusted subsequently to 6 mg to maintain international normalized ratio between 2 and 3. Enoxaparin was stopped after the international normalized ratio reached the desirable range, and warfarin was continued for 3 months.

## Discussion

Several mechanisms contribute to an increased susceptibility to secondary infections following dengue viral infections. One such mechanism is endothelial dysfunction that allows bacteria to invade tissues. Anti-nonstructural protein 1 (anti-NS1) antibodies cross-react with endothelial cells and trigger intracellular signaling, leading to production of nitric oxide and to apoptosis [10]. When this type of damage occurs in the endocardium, bacteria can lodge in the damaged endocardium and cause endocarditis.

Another mechanism is defective functioning and reduction in number of cells of the immune system. Dengue virus infects and replicates in dendritic cells, the primary sentinels of the immune system, and blocks interferon (IFN)-α/β signaling [11]. Antigen-presenting cells isolated from patients with acute dengue infection show defects in T cell priming [12]. Leukopenia, particularly affecting neutrophil and monocyte lineages, is well described and is thought to be related to bone marrow suppression [13]. In the bone marrow, early blast cells are infected by dengue virus and, thus, are phagocytosed by dendritic cells. Infected reticular cells of the bone marrow stroma fail to support hematopoiesis [14].

Alteration of cytokines causing immune dysregulation is another mechanism that contributes to the increased susceptibility to secondary infections. For example, plasma levels of interleukin-10, which is a known immunosuppressant, are increased in dengue infection [15].

Studies have shown that patients with dengue hemorrhagic fever who have prolonged fever (more than 5 days) and acute kidney injury are at high risk for concurrent bacteremia [16]. These factors may have predisposed our patient to develop infective endocarditis.

*Staphylococcus aureus* is found in skin flora and can enter circulation through a breach in the skin. In our patient, skin breaches occurred during insertion of femoral venous catheter for hemodialysis, cannulation for intravenous fluid administration, and repeated drawing of blood for frequent blood tests, which may have facilitated entry of skin flora and resultant bacteremia. This bacteremia occurring against a backdrop of immunosuppression resulting from dengue virus infection may have promoted infective endocarditis in a patient with previously normal heart valves.

Several mechanisms are described in the literature for thrombosis occurring in conjunction with dengue viral infection. Dengue virus interferes with the components of the anti-clotting pathway. Dengue virus downregulates thrombomodulin-thrombin-protein C complex formation, leading to a reduction in activated protein C [17]. Low concentrations of plasma anticoagulant proteins C and S and antithrombin III are detected in severe dengue. The levels decrease with increasing severity of shock, probably due to capillary leakage [18].

Dengue virus activates endothelial cells and increases the expression of thrombomodulin [17].

Plasminogen activator inhibitor type-I (PAI-1) plasma concentrations are elevated in dengue infection [19].

Prolonged shock in dengue shock syndrome may trigger or accelerate the development of disseminated intravascular coagulation and consequent microthrombi formation, but it has not been associated with large vessel thrombosis [20]. Antiphospholipid antibodies and increased lupus anticoagulant are anecdotally associated with thrombotic events in patients with dengue viral infections [9, 21].

Thus a myriad of factors may predispose patients with dengue viral infections to develop thrombotic complications. In addition, endothelial damage due to insertion of femoral venous catheter for hemodialysis may have further increased the risk of deep vein thrombosis in our patient.

## Conclusions

This case illustrates the need to be aware of the possibility of the occurrence of serious secondary bacterial infections following dengue viral infection, especially in patients with prolonged fever (more than 5 days) and acute kidney injury, and highlights the possibility of thrombosis occurring as a rare complication of dengue viral infection.

## Abbreviations

DF: Dengue fever
MRSA: Methicillin-resistant *Staphylococcus aureus*
